# Cross-Cultural Comparison of Self-Construal and Well-Being between Japan and South Korea: The Role of Self-Focused and Other-Focused Relational Selves

**DOI:** 10.3389/fpsyg.2017.01516

**Published:** 2017-09-05

**Authors:** Joonha Park, Vinai Norasakkunkit, Yoshi Kashima

**Affiliations:** ^1^Management, Nagoya University of Commerce and Business Nisshin, Japan; ^2^Psychology, Gonzaga University Spokane, WA, United States; ^3^Psychological Sciences, University of Melbourne Parkville, VIC, Australia

**Keywords:** relational self, self-construal, Koreans, well-being, Japanese

## Abstract

Relational self, along with individual and collective selves, is a fundamental aspect that makes up self-concept. Proposing its two aspects: *self-focused relational self* (i.e., perceiving the self as the object of other people's referential awareness or intentionality) and *other-focused relational self* (i.e., perceiving the self as being attuned and empathetically connected to close others), the current study explored the way the four selves affect well-being in Japan and South Korea, the East Asian cultures that have been assumed to be homogeneously collectivistic in previous psychological literature. Japanese and Korean participants rated a set of well-being and self-related scales. There were visible sample differences within culture by collection method (classroom vs. online) in relative degrees of selves and related constructs, possibly associated with generational differences. Other-focused relational self was greater in the Korean classroom sample than the Japanese counterpart, whereas no difference was found between the online samples. On the other hand, it was consistent between cultures that the two types of relational self showed different associations with social anxiety and self-esteem as expected, and that they predicted well-being in different ways. We discuss implications for the generational differences and their interactions with culture and the importance of separating the two aspects of relational self in the study of self and culture.

## Introduction

People from different cultures tend to construe themselves in different ways. Markus and Kitayama's (1991) original proposals of two distinct ways, independent self and interdependent self, made a dramatic impact on the study of culture and self, illuminating cultural diversity in how the self is construed in relation with others (i.e., autonomy-oriented vs. relatedness-oriented). Despite the great contribution, however, the dichotomous frame between independence and interdependence has also been controversial mainly for its simplicity that it often fails to capture more dynamic aspects of self-construal depending on contexts (e.g., Vignoles et al., 2016). Considering multifaceted aspects of self-construal would benefit from elaboration of culture-specific psychologies between supposedly similar cultures (e.g., differences within East Asian cultures, Anglo European cultures, African cultures, etc.). In the current study we focus on three types of self-construals. As hinted in previous research (Kashima et al., 1995), some of them may better reflect different characteristics between two East Asian cultures, namely, Japan and S. Korea.

We focus on the idea that people pursue and achieve self-definition in terms of their personal, relational, or group characteristics. The three fundamental aspects correspond to self-concepts of individual self, relational self, and collective self, respectively. According to Sedikides et al.'s (2011) description, the individual self reflects one's unique side consisting of attributes that differentiate the person from others, the relational self reflects one's interpersonal side consisting of attributes that are shared with close others, and the collective self is about one's intergroup side consisting of attributes shared with ingroup members and differentiate the ingroup from outgroups. In Kashima and Hardie (2000), prototypical individualists are described as those who define themselves in terms of their unique characteristics” (e.g., physical or personality characteristics or interests) independent from others. These people believe that in the long run, the only person they can count on is themselves. To them, it is very important to make themselves happy in their life. Prototypical relationalists are those who define themselves in terms of their relationships with others (e.g., with family and close friends). They believe that the biggest drive in their life is connectedness with their family and friends. To them, it is very important to maintain interpersonal harmony with their family members and friends. Lastly, prototypical collectivists are said to be those who define themselves in terms of the social groups they belong to (e.g., nationality, occupation). They believe that belonging to their university/company and their country is the most important thing in their life. To them, it is very important to meet their obligations and duties, keep social norms and achieve group goals.

Compared to the individual and collective selves though, the relational self has received relatively less attention in cultural and social psychological research (Kashima et al., 1995; Cross, 2009). Related to universal need to belong (Baumeister and Leary, 1995), one's self-construal in relation with significant, close, or familiar others is deeply connected to an understanding of fundamental humanity (Park et al., 2011). An increasing number of researchers have pointed out its conceptual distinction from the two other kinds of self-construals. In particular, whereas collective self reflects a self-definition in terms of one's memberships in groups or social categories, relational self reflects a self-definition in terms of ties with close or specific others in interpersonal contexts (Kashima and Hardie, 2000; Brewer and Chen, 2007). Interdependent self discussed in much of the literature (e.g., Hashimoto and Yamagishi, 2013) is compatible with collective self, as this type of self reflects a self-definition in terms of anonymous large collectives emphasizing group memberships. Indeed, the two concepts, relational self and collective self (or interdependent self), are often confused in the literature especially when they are juxtaposed against the individual self (Sedikides and Brewer, 2001).

The purpose of the present study is mainly two-fold. First, it attempts to distinguish different aspects of the relational self, as well as re-examine whether the degrees of Japanese and South Koreans (Koreans, hereafter) are similar regarding a particular form of relational self, as suggested by Kashima et al. (1995), but in an extended range of samples in both cultures. Kashima et al.'s (2011) review brings Japan as a particular case where the affective (vs. cognitive) aspect of relational self has been downgraded after World War II. The authors, however, admit that its generalization to other Asian cultures is unwarranted given the higher degree of relational self observed among Koreans than among the Japanese (Kashima et al., 1995).

Second, it aims to examine how different aspects of self-construals predict well-being. Individuals' subjective well-being or life satisfaction is an important issue in contemporary societies and is understood to have significant relationships with personality characteristics reflecting particular aspects of self-concepts. A few studies have considered cultural differences and similarities in predictors of subjective well-being, and there are at least three different perspectives. First, some have found that individual self is a cnsistent predictor of well-being. Diener et al.'s (1995) findings from a large university student sample across 55 nations support that individual self or individualism and wealth at both national and individual levels are strong predictors of subjective well-being across countries.

Also, there is a suggestion that collective self may have a significant effect on well-being especially in Asia. There is cross-cultural evidence that people with personalities that match the dominant cultural norms of their society experience higher well-being than those with unmatching personalities, implying a positive relationship between well-being and interdependence of individuals in East Asia regarding the cultural norm of high collectivism or interdependence (“person-culture match effect,” Fulmer et al., 2011, see also Kitayama et al., 2006). Similarly, Kwan et al. (1997) found that relationship harmony, a construct emphasized in collectivistic cultures, has a significant effect on life satisfaction across cultures, with some cultural difference in the importance of each value. They found that the relative importance of relationship harmony to self-esteem in predicting life satisfaction was greater in the collective culture of Hong Kong than in the individualistic culture of the United States (Study 1).

To our knowledge, however, little is known regarding the relationships between well-being and relational self, compared to the well-explored relationship between individual self and well-being. In fact, the relationship harmony in Kwan et al.'s (1997) work is rather seen as a form of relational self than collective self which is often related to large collectives. This suggests the importance of examining contributions of relational self to one's well-being in addition to the two primary selves. To sum up, examining whether multiple aspects of self, including the proposed relational self, play different roles in predicting life satisfaction would shed further light on relationships between self-construal variables and well-being across cultures.

### Relational self-construal

Given that relational self involves interpersonal relationships between the self and other(s), there can be at least two types of relational self, depending on the focus (self-focused vs. other-focused). In *self-focused relational self*, one is mainly concerned about the way the other(s) view, evaluate, and accept or reject the self as a desirable counterpart. This aspect can be associated with one's need for positive self-image in interpersonal relations (Crocker and Canevello, 2008). Kashima et al.'s (2011) cognitive relational self corresponds to this type of relational self. To borrow their description about cognitive relational self, *self-focused relational self* is primarily concerned with “referential self-awareness, a person's awareness of the fact that he or she is an object of other people's referential awareness or intentionality (p. 16).” Thus, a person with high *self-focused relational self* is very conscious of how he or she is perceived and evaluated by others. This type of relational self overlaps with some components of social anxiety, such as the fear of being negatively evaluated by others. Thus, it is plausible to expect that those with higher social anxiety levels as a default emotion in social contexts would be more likely to harbor a self-focused relational self. Although self-focused relational self may overlap with the cognitive component of social anxiety (i.e., evaluation apprehension) and some emotional component of social anxiety (i.e., fear), it is not necessarily associated with the behavioral component of social anxiety (i.e., avoidance of social situations; Beidel et al., 1985). Therefore, self-focused relational self is not about being socially anxious even though some aspects of it may be close to social anxiety.

To understand the nature of self-focused relational self, it is worth reviewing Takata's (2000) two aspects of interdependent self-construal: fear of reputation and adaptation to others. Although, Takata was not clear about whether the interdependent self captures relational self, collective self, or both, we regard that his concept is mainly relevant to the former, as the items developed in his research are concerned more with self-construal in specific interpersonal relations than in group contexts (Kashima and Hardie, 2000). Indeed, when analyzed with Kashima and Hardie's RIC (relational, individual, and collective self-construal) scale, the Takata items were not correlated with collective self but with relational self. In particular, Takata's fear of reputation items seem to mostly cover self-focused relational self, where one's referential self-consciousness is highly involved (i.e., “I am concerned about what people think of me,” “Sometimes when I do things I get so anxious and confused thinking about how everything will turn out, that I have trouble even getting started”). Therefore, we consider Takata's fear of reputation concept as corresponding to *self-focused relational self*.

On the other hand, with the *other-focused relational self*, one's primary concern is about being empathically attuned with and worrying about specific other(s). This aspect may be related to sympathetic or compassionate goals in interpersonal relations (Crocker and Canevello, 2008). Although *self-focused relational self* is related to one's concern for others too, it is more about one's consciousness and worries about others' perception of self. It is clearly different from *other-focused relational self*, where the self is motivated to empathize with the other person's feelings and thoughts. Closely related to this is Kashima et al.'s (2011) affective relational self, where one's own affective or non-verbal relatedness or empathetic connectedness with others is the most prominent thing. Kashima and Hardie's (2000) relational self scale captures this aspect well (e.g., “I often feel sorry for people who look lonely in a gathering and try to talk with them,” “I try to put myself in other people's shoes”). It is worth clarifying here that interpersonal goals and the two types of relational self are conceptually different. Relational self refers to the way one construes the self in close interpersonal relations, and is not the same thing as the specific goals one tries to pursue in interpersonal relationships, although those with relational selves may often be motivated by specific interpersonal goals.

As suggested in Kashima et al. (1995), Koreans more than the Japanese tend to be higher on the affective part of relational self. Since Japanese colonization, followed by the Korean War in 1950, Korea had been faced with dynamic national situations where many people moved around the country or out of the country for political or economical reasons, resulting in many refugees and separated families, which is still perceived as a national tragedy. Such migratory experiences, causing unwanted breakups among family members, could have made kinship especially precious to the people. Kim (2002) explains that the violent governmental system since the colonization period and the process of modernization lacking opportunities to experience the nation as a contractual entity have resulted in strong familism in which citizens put priority on devotion to family, or family-like relationships over devotion to the country or society. The strong emphasis on family persists to this day despite influences of Western style individualism (Suh, 2003). Even non-familial relationships often resemble a familial structure in interpersonal contexts where emotional attachment is a key element in understanding characteristics of Korean people's interpersonal relationships (Choi, 2011). We speculate that such familial devotion and emphasis on emotional attachment in social contexts are closely related to relational self, especially *other-focused relational self*.

### Hypotheses of the current study

The study aimed to distinguish the two types of relational self in juxtaposition to other relevant constructs. First, we expected that cultural difference would exist on the relative degrees of each type of relational selves. Based on the previous finding that Japanese and Koreans are not homogeneous in relational self (Kashima et al., 1995), the current study aimed to examine relative degrees of different selves within the specified construct. The researchers adopted *Kanjin-shugi* (“between-people-ism”) scale for measuring relational self, which was supposed to tap into the content that generally emphasizes emotional relatedness of the self with other individuals (Kashima et al., 1995). Given the conceptual relationship with the previous scale, it was hypothesized that Koreans would be higher on particularly *other-focused relational self* than Japanese (H1). We did not make specific predictions for differences in other self constructs between two cultures.

As described earlier, the different forms of relational selves would involve different degrees of social anxiety in one's interpersonal experiences. It was hypothesized that social anxiety would be positively correlated with self-focused relational self and negatively with other-focused relational self (H2). Next, there seem to be different relationships between the two types of relational self and self-esteem. Crocker and Canevello's (2008) study on interpersonal goals and well-being shows that those with higher self-image goals have lower self-esteem and experience less well-being compared with those with higher compassionate goals (see also Kwan et al., 1997). Self-image goals and compassionate goals may be associated with *self-focused relational self* and *other-focused relational self*, respectively. Therefore, we hypothesized that *self-focused relational self* would have a negative relationship with self-esteem, whereas *other-focused relational self* would have a positive relationship with self-esteem, regardless of culture (H3).

Finally, this study examined relationships between various self-concepts and psychological well-being. In particular, it focused on predictions of the two aspects of relational self on well-being between Japan and Korea. It was hypothesized that *self-focused relational self* and *other-focused relational self* would take different forms in predicting well-being. Specifically, cognitive well-being would be predicted negatively by the former and positively predicted by the latter form of relational self in both cultures (H4a). Similar patterns would be found for affective well-being (H4b). Additionally, we aimed to replicate significant effects of individual self and collective self on well-being in general based on the previous studies (Diener et al., 1995; Fulmer et al., 2011).

## Method

### Participants

There were two recruitment methods used in this study. First, 78 Japanese undergraduates at Kansai University (male 59.5%, mean age = 20.97 years old, *SD* = 1.22) and 108 Korean undergraduate at Inha University (male 54.6%, mean age = 20.63 years old, *SD* = 2.4) participated in the survey in a lecture setting under supervision of research assistants at each university. Second, 110 Japanese (male 51.6%, mean age = 45.26 years old, *SD* = 14.25) and 115 Korean participants (male 48.7%, mean age = 36.90 years old, *SD* = 9.49) were recruited through data collection companies in each country and participated in the same survey online. As all participants rated on the same set of items, despite the difference in methods of collection, the data sets were all combined, resulting in ratings of a total of 185 Japanese and 223 Korean participants. Full review and ethics approval were not required for this study according to the institutional guidelines in each culture.

### Materials and procedure

Participants filled out a questionnaire prepared in their native languages (Japanese or Korean). It involved a set of standard well-being, self-construal, social anxiety and self-esteem scales as follows. Original versions of each scale were in English, but then translated into Japanese by one English-Japanese bilingual, and it was checked by back-translation by another English-Japanese bilingual. The same procedure was used for Korean.

### Subjective well-being and self-esteem

In that subjective well-being can be both felt (affective) and perceived (cognitive), we prepared two well-being scales to capture each of the aspects, Scale of Positive and Negative Experiences (SPANE, 12 items, Diener et al., 2010) and Satisfaction With Life Scale (SWLS; five items, Diener et al., 1985, respectively) of well-being. This distinction has been typical in well-being research and a few studies have adopted both scales to measure subjective well-being (e.g., Bastian et al., 2012). We also included Rosenberg's self-esteem scale (10 items) to test distinct relationships with two aspects of relational self.

### Individual, collective, and relational self-construal

Singelis (1994) 24 items of independence and interdependence were used for measuring individual and collective selves, respectively. There is a strong emphasis on the ingroup in his interdependence scale, which is considered to tap collective self (e.g., “It is important to me to respect decisions made by the group;” Kashima and Hardie, 2000). Takata's (2000) four items of fear of reputation (e.g., “I am concerned about what people think of me”) were used for measuring *self-focused relational self*. Kashima and Hardie's (2000) seven items of relational self scale were used for measuring *other-focused relational self* (e.g., “I try to put myself in other people's shoes”), because the individual in those items is motivated to focus on close others and be attuned with them. Six items measuring social anxiety (Fenigstein et al., 1975, e.g., “It takes me time to get over my shyness in new situations,” “I get embarrassed very easily,” “Large groups make me nervous”) were included to examine if the two types of relational self are correlated with social anxiety in different ways. Participants rated all items on 7-point scale.

## Results

We standardized participants' responses across main variables within each participant to remove response sets such as acquiescence responding style and extreme responding style (within-subject standardization). In this procedure, the mean and standard deviation of each participant's responses to a given questionnaire are computed and used to transform raw scores into standard scores (Fischer and Milfont, 2010).

### Cultural comparisons on self-related dimensions

Table 1 shows reliability of self-construal, self-esteem, and social anxiety scales measured and their overall mean values in each culture and collection method. A 3-way mixed measures ANCOVA was conducted with a set of four self constructs as a within-subject factor, culture and collection method as between-subject factors, and age as a covariate, to examine effects of culture, collection method and age on mean values of different self constructs. Mauchly's test indicated that sphericity was not assumed, χ^2^_(5)_ = 138.13, *p* < 0.001. The following results are based on Huynh-Feldt correction, as Greenhouse-Geisser Epsilon was 0.81, which was above the criterion value (0.75, Howell, 2002). There was a self main effect, *F*_(2.45, 998.71)_ = 13.52, *p* < 0.001, as well as an interaction effect between self and age, *F*_(2.45, 998.71)_ = 4.59, *p* < 0.001, indicating that dominant self-construals varied across age. Interaction between self and culture was also significant, *F*_(2.45, 998.71)_ = 3.17, *p* = 0.03, indicating that there was a cultural difference in ratings on the four self constructs. Also, there was a marginal interaction effect between self and collection method, *F*_(2.45, 998.71)_ = 2.28, *p* = 0.09, and also a significant three-way interaction between self, culture, and collection method, *F*_(2.45, 998.71)_ = 3.55, *p* = 0.02. All these imply that ratings on different selves were different depending on age, culture, collection method and interaction between culture and collection method. It is noteworthy that the factor of collection method was interacted with age in our data, as implied in the large differences in average age between classroom and online samples (i.e., more than 15 years old gap in both countries). Thus, we concluded that it would be proper to conduct further analyses separately for each collection method in order to test H1.

**Table 1:** Reliabilities and means of main variables for each culture (Japan, Korea) and collection method (classroom, online).

**Variables**	**Japan**			**Korea**		
	***a***	**Mean, *SD* (classroom)**	**Mean, *SD* (online)**	***a***	**Mean, *SD* (classroom)**	**Mean, *SD* (online)**
Other-focused relational self	0.87	−0.14, 0.68	−0.56, 0.61	0.72	0.21, 0.59^***^	−0.05, 0.55
Self-focused relational self	0.82	0.15, 0.98	−0.33, 0.75	0.79	0.22, 0.76	0.01, 0.61
Individual self	0.76	−0.20, 0.62	−0.12, 0.48	0.81	0.26, 0.47^***^	0.004, 0.53
Collective self	0.78	−0.24, 0.64	−0.11, 0.52	0.76	0.17, 0.45^***^	0.11, 0.50^**^
Cognitive well-being	0.90	−0.38, 0.76	−0.22, 0.84	0.89	0.42, 0.72^***^	0.08, 0.83^*^
Affective well-being	0.88	0.04, 0.68	−0.05, 0.55	0.89	0.27, 0.64	−0.24, 0.40
Self-esteem	0.86	−0.37, 0.77	−0.14, 0.65	0.83	0.32, 0.48^***^	0.08, 0.59^**^
Social anxiety	0.87	0.31, 0.85^***^	0.01, 0.71	0.88	−0.27, 0.79	0.03, 0.73

Scores are within-participant standardized. Significant differences between cultures in each of the collection method comparisons examined by series of ANCOVA are indicated with asterisks,

*** p < 0.001,

** p = 0.001,

* *0.001 < p < 0.0125*.

A series of of one-way ANCOVA were conducted with culture as a between-factor for each of the self variables, controlling for age. Bonferroni adjusted alpha levels of 0.0125 per test (0.05/4) were used.

Results showed that H1 was supported in classroom samples, where other-focused relational self was greater among Korean students than Japanese students, *F*_(1, 184)_ = 13.04, *p* < 0.01. However, there was no difference between the Japanese online sample and the Korean online sample, *F*_(1, 222)_ = 0.36, *p* = 0.55. There was no main effect or interaction involving age.

For individual self, the Korean classroom sample scored higher than the Japanese classroom sample, *F*_(1, 184)_ = 35.15, *p* < 0.001. The online samples showed marginally significant cultural differences as well, *F*_(1, 222)_ = 6.02, *p* = 0.02. Again, there was no main effect or interaction involving age.

For collective self, both the classroom Korean sample and the online Korean sample scored higher than their Japanese counterparts, *F*_(1, 184)_ = 26.95, *p* < 0.001, *F*_(1, 222)_ = 10.72, *p* = 0.01. Again, there was no main effect or interaction involving age.

For self-focused relational self, Koreans scored higher than the Japanese at marginally significant levels, but only with the online samples, *F*_(1, 222)_ = 5.56, *p* = 0.02. There was no difference between classroom samples. However, there were significant age main effects in both classroom and online comparisons, *F*_(1, 184)_ = 18.70, *F*_(1, 222)_ = 14.95, respectively, both *p*s < 0.001.

Additional analyses of the similar series of ANCOVA on cognitive well-being, affective well-being, self-esteem, and social anxiety with bonferroni adjustment were conducted. Significant cultural differences on the target variables are indicated in Table 1. To summarize the visible differences, Koreans scored higher than Japanese on cognitive well-being and self-esteem across collection methods. Japanese scored higher than Koreans on social anxiety in the classroom sample only. There was no cultural difference in social anxiety levels for the online samples. Additionally, no cultural differences were found in affective well-being.

In all of the following analyses, our reports are based on analyses of decultured scores for all variables, which are generated by standardizing the within-subject standardized scores within each culture (Leung and Bond, 1989; Kashima et al., 1995). As one of the most common types of transformations used in cross-cultural psychological research (for review, Fischer, 2004), this double-standardization method allows researchers to control for group differences in means and standard deviations of questionnaire items affected by cultural responding style in addition to removing individual-level response sets (Fischer and Milfont, 2010).

Given the meaningful age/generational effects in some of the previous analyses, we additionally conducted correlational analyses for all dependent variables in each culture after aggregating ratings by different collection methods (see Table 2). In Korea, there were generally negative correlations between age and several variables—older generations scored lower on individual self, other- focused relational self, self-focused relational self, cognitive well-being, and affective well-being. They also tended to have lower self-esteem. Japanese samples showed different patterns. Although they similarly showed negative correlations between age and self-focused relational self, social anxiety was also negatively correlated, and more importantly, age was positively correlated with individual self and self-esteem. These unexpected culture by age interaction effects are thought-provoking and difficult to interpret. We will revisit this issue in the Section Discussion.

**Table 2:** Correlations between main variables in each culture.

**Country**	**Variables**	**1**	**2**	**3**	**4**	**5**	**6**	**7**	**8**	**9**
Korea	1	1								
	2	−0.142^*^	1							
	3	−0.004	0.442^**^	1						
	4	−0.162^*^	0.368^**^	0.509^**^	1					
	5	−0.215^**^	0.054	0.265^**^	0.344^**^	1				
	6	−0.126^†^	0.492^**^	0.360^**^	0.337^**^	−0.054	1			
	7	0.053	−0.291^**^	0.074	−0.036	0.451^**^	−0.368^**^	1		
	8	−0.165^*^	0.432^**^	0.336^**^	0.262^**^	0	0.614^**^	−0.175^**^	1	
	9	−0.265^**^	0.419^**^	0.276^**^	0.270^**^	−0.101	0.573^**^	−0.333^**^	0.598^**^	1
Japan	1	1								
	2	0.141^*^	1							
	3	0.079	0.200^**^	1						
	4	0.122^†^	0.341^**^	0.445^**^	1					
	5	−0.343^**^	−0.108	0.312^**^	0.134^†^	1				
	6	0.233^**^	0.377^**^	0.125	0.323^**^	−0.349^**^	1			
	7	−0.202^**^	−0.348^**^	0.257^**^	−0.096	0.510^**^	−0.484^**^	1		
	8	0.132	0.271^**^	0.252^**^	0.207^**^	−0.074	0.639^**^	−0.223^**^	1	
	9	0.013	0.216^**^	0.161^*^	0.191^**^	−0.147^*^	0.528^**^	−0.200^**^	0.530^**^	1

0.05 <

† p < 0.10,

* p < 0.05,

** p < 0.01,

*** *p < 0.001. Scores are double-standardized. 1, Age; 2, Individual self; 3, Collective self; 4, Other-focused relational self; 5, Self-focused relational self; 6, Self-esteem; 7, Social anxiety; 8, Cognitive well-being; 9, Affective well-being*.

### Two aspects of relational self, social anxiety, and self-esteem

As expected, social anxiety was correlated with self-focused relational self only (*r* = 0.49, *p* < 0.001) and not with other-focused relational self (*r* = −0.06, *p* = ns). These patterns were replicated when the cultures were examined separately (Table 2). In support of H2, the implication here is that social anxiety-like behaviours may be an important discriminant factor between focus orientations (i.e., self-focused vs. other-focused) in interpersonal relations.

Similarly, self-focused relational self was negatively and other-focused relational self was positively correlated with self-esteem across cultures (*r*s = −0.20 and 0.33, respectively, both *p*s < 0.001). Again, we found similar patterns even when correlations were examined in each culture (Table 2). All these findings support H3 which states that the relational self, depending on the focus orientation (i.e., self vs. other) in interpersonal relations, has opposite associations with self-esteem.

### Predictions of self dimensions on well-being in both cultures

Finally, we were interested in the way the two relational selves along with individual and collective selves predict cognitive and affective aspects of well-being. Two standard multiple regression analyses were conducted to examine culture (Japan or Korea), individual self, collective self, and self-focused and other-focused relational selves as well as the interactions between different selves and culture as predictors of well-being (affective, SPANE and cognitive, SWLS; Table 3). Due to possible age effects on predictions, age was controlled for in both analyses. In the model predicting cognitive well-being, *R*^2^ = 0.42, *F*_(10, 401)_ = 8.89, *p* < 0.001, individual self and collective self predicted cognitive well-being, βs = 0.26 and 0.21, respectively, both *p*s < 0.001. In partial support of H4a, self-focused relational-self inversely predicted cognitive well-being, β = −0.13, *p* = 0.01, whereas other-focused relational self did not predict well-being, β = 0.07, *p* = ns. There were no significant interaction effects between either type of self and culture, implying that the effects of the predictors were similar between cultures.

**Table 3:** Standardized regression coefficients for four selves, and their interaction terms with culture in multiple regression analyses to predict cognitive well-being and affective well-being.

	**Cognitive well-being**	**Affective well-being**
R^2^	0.18	0.20
F_(10, 400)_	8.89^***^	10.17^***^
Age	−0.04	−0.18^***^
Country	0.01	0.04
Individual	0.26^***^	0.22^***^
Collective	0.21^***^	0.16^**^
R_other	0.07	0.14^*^
R_self	−0.13^*^	−0.26^***^
I × c	−0.05	−0.08
C × c	0.03	0.02
R_other × c	−0.01	−0.02
R_self × c	−0.03	−0.01

Scores are double-standardized. I or Individual, individual self; C or Collective, collective self; R-self, self-focused relational self; R-other, other-focused relational self; c, culture (−0.5 = Korea, 0.5 = Japan);

* p < 0.05,

** p < 0.01,

*** *p < 0.001*.

In the model predicting affective well-being, *R*^2^ = 0.20, *F*_(10, 400)_ = 10.17, *p* < 0.001, there was an age effect, β = −0.18, *p* < 0.001, implying that affective well-being was inversely associated with age. Similar to the previous model, individual self and collective self predicted affective well-being, βs = 0.22 and 0.15, *p* < 0.001, and *p* < 0.01, respectively. Consistent with H4b, other-focused relational self was positively associated with affective well-being, β = 0.14, *p* = 0.01, whereas self-focused relational self was inversely associated with affective well-being, β = −0.26, *p* < 0.001. Again, there were no significant interaction effects between types of self and culture.

## Discussion

The current study provides overall supports for our hypotheses despite some unexpected observation in collection method differences. First, other-focused relational self was greater among Korean student sample than the Japanese counterpart, in partial support of H1, and also consistent with the previous finding (Kashima et al., 1995). However, no cultural difference was observed in online samples. Second, social anxiety was positively correlated with self-focused relational self but not with other-focused relational self, partially supporting H2. Similarly, self-esteem was positively correlated with other-focused relational self and negatively with self-focused relational self in support of H3. Finally, H4a was partially supported as self-focused relational self negatively predicted cognitive well-being although other-focused relational self did not predict it. On the other hand, H4b for affective well-being was fully supported as it was predicted positively by other-focused relational self and negatively by self-focused relational self. These findings provide convincing evidence for different motivations and implications of the two relational selves in interpersonal relations, positive self-regard and well-being.

### Cross-cultural comparisons between Japan and Korea

The lack of cultural difference on other-focused relational self in the online samples is inconsistent with previous findings (see Kashima et al., 1995). However, it may not be surprising if we are reminded that the target samples in the previous study were limited to college students in different cultures, whereas most of our participants in online collection method were non-students. Significant age effects on the overall ratings on self-related variables, as well as correlations between age and the self variables including the other-focused relational self, imply that the inconsistencies between collection methods probably represent generational differences.

The correlational results are especially noteworthy. First, the negative relationship between age and other-focused relational well-being in Korea may explain why Kashima et al.'s (1995) previous finding was replicated only in the present classroom (student) sample but not in the online (adult) sample. The pattern among older generations is surprising given the historical and cultural background reflecting the importance of familial attachment as described earlier in this article. One possible explanation may be related to the cultural norms for emotional expressions in interpersonal contexts. Even within a close relationship, Korean older generations may feel less comfortable with expressing their feelings and empathy toward the other. Such norms with respect to emotional expression may limit the construal of self as other-focused for Korean older generation. Indeed, the repression of emotional expressions seems to be associated with culture-bound physical symptoms such as *hwa-byung* (literally meaning “illness of anger”) among older generations (Choi et al., 2016). Future research should investigate Korean people's self-construal and well-being across generations to examine whether there are any generational differences with respect to emotion display rules in interpersonal context and how this is associated with well-being related variables.

Moreover, a similar correlational pattern for the relationship between age and individual self in Korea suggests that such generational differences also exist in other self-related domains. This is not the first finding though, as noticed in previous research (Inglehart, 1997; Na and Duckitt, 2003). According to Ingelhart's study based on a survey of 43 industrialized countries, Korea showed the greatest generation gap, so that both modern and postmodern values coexist in the country depending on generations. Similar to the previous findings, Korean older generations in our study tended to be more or less consistent with the traditional view of Asians (i.e., lower levels of individual self, self-esteem, and well-being relative to the younger generations). Thus, future research can also more generally examine generational differences in how well-being related measures are associated with attitudes toward traditional norms and cultural change in current Korean society.

While older generations of both Koreans and Japanese were similarly less apprehensive of other people's evaluations of them, Japanese older generations were different from the Korean older generation in some interesting ways. Relative to the younger generation, Japanese older generations appeared to be rather more individualistic, less anxious in social contexts, and thus experience higher subjective well-being in general. This Japanese pattern is consistent with Takata's (2004) analysis on developmental change in relative degrees of in(ter)dependence suggesting that the independent self develops after adulthood in Japanese culture whereas interdependent self is internalized in adolescence.

In understanding the case of Japanese youth, on the other hand, it is noteworthy to look at Norasakkunkit and Uchida's (2011) recent argument about a social psychological phenomenon among Japanese youth in post-industrialized Japan. According to the authors, nowadays many Japanese youth are in the state of *anomie* because they are being increasingly excluded from occupying more secure social/economic spaces in Japanese society due to exclusionary institutional reactions that protect the senior elites at the cost of marginalizing younger people in the core labor markets. As a result, many young Japanese adults have neither adopted the values of another culture (e.g., Western culture) nor fully embraced indigenous, mainstream cultural values of interdependence, thereby leading to a kind of marginalization in which both the individual self and the collective self are relatively diminished. In combination with Takata's (2004) developmental analysis, the data suggest that Japan has a significant generation gap in which the older adults have more agency (both independent and interdependent) and higher degrees of well-being compared to the younger generation (see also Grossmann et al., 2014). In contrast, well-being for older Koreans are generally lower than that of younger Koreans today. Furthermore, agency, as measured by most of the self variables (individual, collective, and other-focused relational), seem to be lower for younger Japanese than for younger Koreans.

The current study suggests that gross generalization about self-construal and their correlates across Asian cultures are unwarranted, as there can be fundamental heterogeneity in their geographical conditions, attitudes toward religion (Norasakkunkit and Uchida, 2012), and history at least since modern times as described earlier (see also Kashima et al., 1995). For example, Norasakkunkit and Uchida points out some subtle difference between Japan and Korea suggesting different roles that religion, especially Christianity, played between the two countries in adapting to post-industrialization and globalization. Whereas Christianity, especially Protestantism, played an important role in affording the type of agency and new value system for the Koreans during the era of uncertainty after WWII (Zielenziger, 2006), religion was sufficiently muted throughout Japanese history, partly due to the strict authority of warrior autocrats (e.g., the Shogun during the Edo period) in combination with the pervasive value of social assurance rather than trust (for review, Norasakkunkit and Uchida). Such society-specific characteristics may have contributed to meaningful heterogeneity across East Asian cultural contexts, especially in today's world where pressures to make structural and ideological adjustments are often exerted from outside the society (i.e., globalization, climate change, etc.). Thus, our study calls for a future investigation with separate and more insightful analyses of self-related constructs and well-being among various generations in each society.

Although, generational effects on self-construals were not the primary focus of the current study, these unexpected findings provide important implications for social and cultural psychology. Specifically, they suggest a strong need to take into account generational effects within cultures on various associations between self-relevant variables and well-being variables. In other words, it seems important to include different cohorts in cross-cultural studies. To our knowledge, there is lack of research that has looked at multi-level analyses that include both generational effects and culture effects (Takata, 2004), especially with respect to how people adapt to cultural change and globalization (Na and Duckitt, 2003).

### Predictions of self-construals on well-being

Our findings illustrate the various ways that self-construal variables predict affective and cognitive well-being across social contexts. Incorporating a general understanding of previous research on well-being in cultural psychological studies (Diener et al., 1995; Fulmer et al., 2011), we found that both of the individual self and the collective self served as powerful predictors of affective and cognitive well-being in East Asia (Japan and Korea).

As expected, the two relational selves also played a role in well-being: *self-focused relational self* in general was associated inversely with well-being, whereas *other-focused relational self* was associated positively with well-being. These findings support the idea that *self-focused relational self* and *other-focused relational self* are associated with self-image goals and compassionate goals respectively (Park and Kashima, 2014). Indeed, Crocker and Canevello's (2008) work shows that self-image goals predict conflict, loneliness, and feeling of fear and confusion, all of which may decrease happiness. In contrast, compassionate goals predict closeness, clear and connected feelings, and increased social support and trust, all of which may increase happiness. The assumed relationships between different interpersonal goals and relational selves are further strengthened by the divergent relationships between the two relational selves and well-being related measures. Specifically, *self-focused relational self* was inversely associated with self-esteem, while *other-focused relational self* was positively associated with self-esteem. Furthermore, these patterns were reversed when self-esteem was replaced with social anxiety. These findings suggest that the two relational selves are associated with the motivational goals of interdependence in different ways (i.e., other-focused relational self being associated with empathic attunement with others; self-focused relational self being associated with fear of negative evaluation). Future studies should experimentally test whether self-esteem and subjective well-being would increase when one's relational self is more concerned about the other than about the self.

Additionally, age appeared to be more involved in experience of affective well-being than cognitive well-being, so that older generations feel less happiness than younger generations. Although tentative, this tendency may be explained by the dominant cultural script in the region that seeks to find a middle way by experiencing a balance between positive and negative emotions in contrast to the West where there is a preference to maximize positive emotions and minimize negative emotions (Kitayama et al., 2000).

Through multiple collection methods and a wide range of age, the current findings provide convincing evidence of the different roles of the two types of relational self on people's well-being. Nevertheless, one crucial limitation is the different age distributions between classroom and online samples, which conflated age effects with collection method effects. The culture by generation interaction effects uncovered in this study have to also be elaborated in future investigations where this interaction can be examined in more depth. Also, although social anxiety is understood to consist of three aspects, emotional (physiological), cognitive, and behavioral (Beidel et al., 1985), the social anxiety measure in the current study did not separate them and some of them tended to tap evaluation apprehension, which is closely related to the self-focused relational self. For this reason, it might be less interesting to look at the relationship between the two concepts. It would be more meaningful for future research to examine relationships between the behavioral aspect of social anxiety (e.g., avoidance of social situations) and the two types of relational self.

Finally, it is necessary to examine the two aspects of relational self in other cultures as well, especially in more individualistic cultures, to see if they are divergently associated with subjective well-being measures. Kwan et al.'s (1997) study implied universal importance of relatedness or relational harmony in promoting one's well-being. Assuming the bi-directional forms of relational self (e.g., self-focused vs. other-focused), future research can further elaborate on the role of more specific self-construals on well-being across cultures.

## Conclusion

The current study was the first to investigate the relationships between the two types of relational self and well-being between Japan and Korea with both classroom and online samples in each culture. Questioning the assumed East Asian homogeneity (e.g., prevalent collectivism, interdependence), the study suggests instead some important variability across specific cultural contexts with respect to the associations between self variables and well-being variables. Also, the study suggests a considerable psychological variance across age groups within East Asian cultures and the importance of studying cultural change across generations in non-Western, post-industrial societies. Nevertheless, the robust findings across cultures and collection methods on the two types of relational self highlight the importance of distinguishing between the two aspects of relational self in the study of the relationship between self-construal and well-being.

## Ethics statement

This study was carried out in accordance with the ethics guidelines of the Japanese and Korean universities and the respective data companies where participants were recruited.

## Author contributions

JP led the study, building up hypotheses, designing survey methods, collecting and analyzing data and writing up the manuscript. VN collaborated by advising on theoretical development, interpretations of analysis results, and editing the manuscript. YK advised on theoretical development and interpretations of the findings.

### Conflict of interest statement

The authors declare that the research was conducted in the absence of any commercial or financial relationships that could be construed as a potential conflict of interest.
